# Supplementary material for: Prevalence and mechanisms of somatic deletions in single human neurons during normal aging and in DNA repair disorders
Source: Nat Commun. 2022 Oct 7;13:5918. doi: 10.1038/s41467-022-33642-w (PMC9546902; doi:10.1038/s41467-022-33642-w)
Supplement: Supplementary file 1 — Supplementary Information [file 41467_2022_33642_MOESM1_ESM.pdf]

Supplementary Information

Prevalence and mechanisms of somatic deletions in single human neurons during normal aging and in DNA repair disorders

Junho Kim[1,2,3,4,5], August Yue Huang[1,2,3,4], Shelby L. Johnson[6], Jenny Lai[1,2,3,4], Laura Isacco[1,2,3,4,7,8], Ailsa M. Jeffries[6], Michael B. Miller[1,2,3,4,7,8,9], Michael A. Lodato[1,2,3,4,6,7,8], Christopher A. Walsh[1,2,3,4,7,8]*, and Eunjung Alice Lee[1,2,3,4]*

[1]Division of Genetics and Genomics, Boston Children's Hospital, Boston, MA, USA

[2]Manton Center for Orphan Disease, Boston Children's Hospital, Boston, MA, USA

[3]Department of Pediatrics, Harvard Medical School, Boston, MA, USA

[4]Broad Institute of MIT and Harvard, Cambridge, MA, USA.

[5]Department of Biological Sciences, Sungkyunkwan University, Suwon, South Korea.

[6]Department of Molecular, Cell and Cancer Biology, University of Massachusetts Medical School, Worcester, MA, USA.

[7]Howard Hughes Medical Institute, Boston Children's Hospital, Boston, MA, USA

[8]Department of Neurology, Harvard Medical School, Boston, MA, USA.

[9]Department of Pathology, Brigham and Women's Hospital, Harvard Medical School, Boston, MA, USA.

*Correspondence: christopher.walsh@childrens.harvard.edu (C.A.W), EAlice.Lee@childrens.harvard.edu (E.A.L)

Supplementary Figures

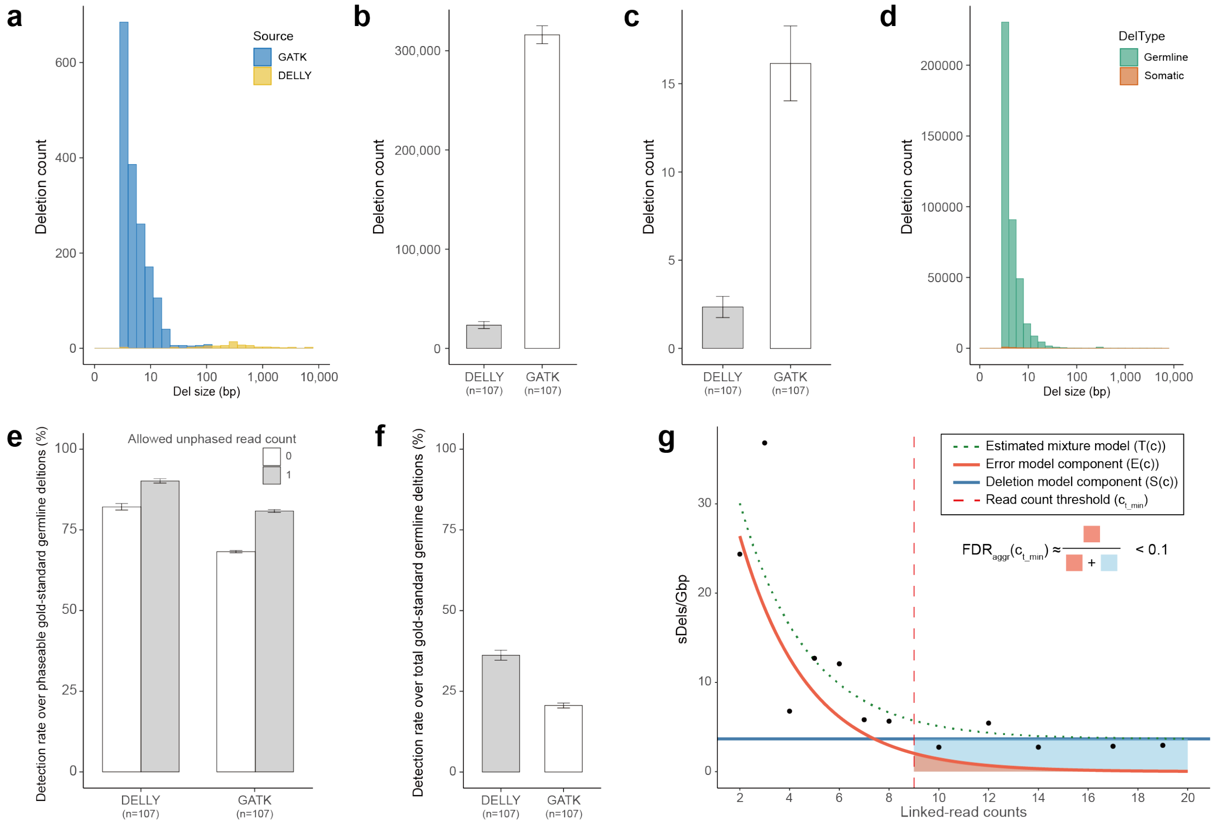

Fig. S1. Characteristics of PhaseDel deletion calls. (a) Histogram for the size distribution of somatic deletions detected by two initial callers, GATK and DELLY2. (b, c) The average of initial (b) and phased (c) deletion counts per cell. The initial deletion set represents raw calls including false positives and germline deletions, and phased deletions are the final somatic calls after the linkage analysis by PhaseDel. (d) Histogram for the size distribution of somatic and germline deletions. (e, f) The fraction of phaseable (e) and total (f) gold-standard germline deletions detected by PhaseDel. Two different fractions with (grey bar) or without (white bar) allowing one unphased read were measured and compared for phaseable regions (e). One unphased read was allowed for measuring the total fraction (f). (g) Estimation of somatic deletion rate using a two-component model. From a given single cell, all phased deletion candidates were grouped into many subgroups based on their linked-read counts between a deletion and a nearby germline heterozygous SNP. The observed rate was calculated for each subgroup that had the same linked-read count (black dots), then a mixture model was fitted for their distribution (*T(c)*, green dotted line) to estimate two components—a true somatic deletion (*S(c)*, blue line) and an error (*E(c)*, red line). Estimated constant for *S(c)* was reported as an estimated somatic deletion rate for a given cell. Based on the fitted model, read count threshold (c_{t_min}, red dashed line) was determined as the minimum read count with FDR_{aggr}<10%, estimated by the fraction of area under two fitted curves. Candidates with the supporting read counts > c_{t_min} were selected as a high confidence set and used for the entire analysis. Detailed description for the estimation of somatic deletion rate is described in Methods. n, number of single neurons; bar graph, mean±95% confidence interval (CI). Source data are provided as a Source Data file.

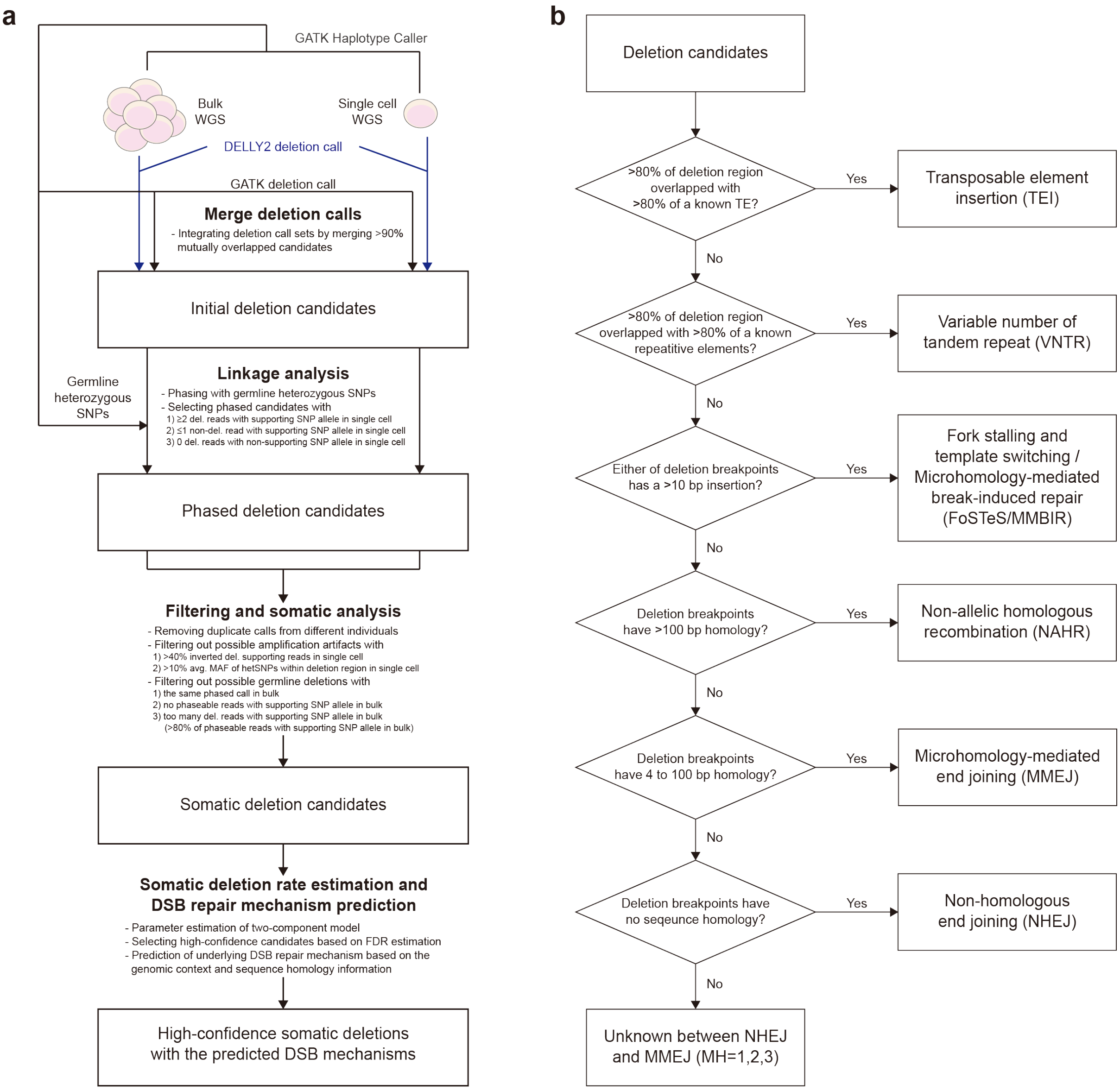

Fig. S2. A schematic flow diagram of PhaseDel. (a) An overview of the PhaseDel workflow. See Methods for detailed description of each step. (b) The flowchart for determining the underlying DSB repair mechanism for deletion candidates. The deletion categories and their classification criteria are adopted from Yang et al.[1] A deletion is classified into six different categories based on the genomic element and sequence homology between the deletion breakpoints. Deletion candidates with sequence homology of 1-3 bp might originate from either NHEJ or MMEJ, therefore they are classified as unknown group (MH=1,2,3) and are excluded from further analyses. TE, transposable element; MH, microhomology.

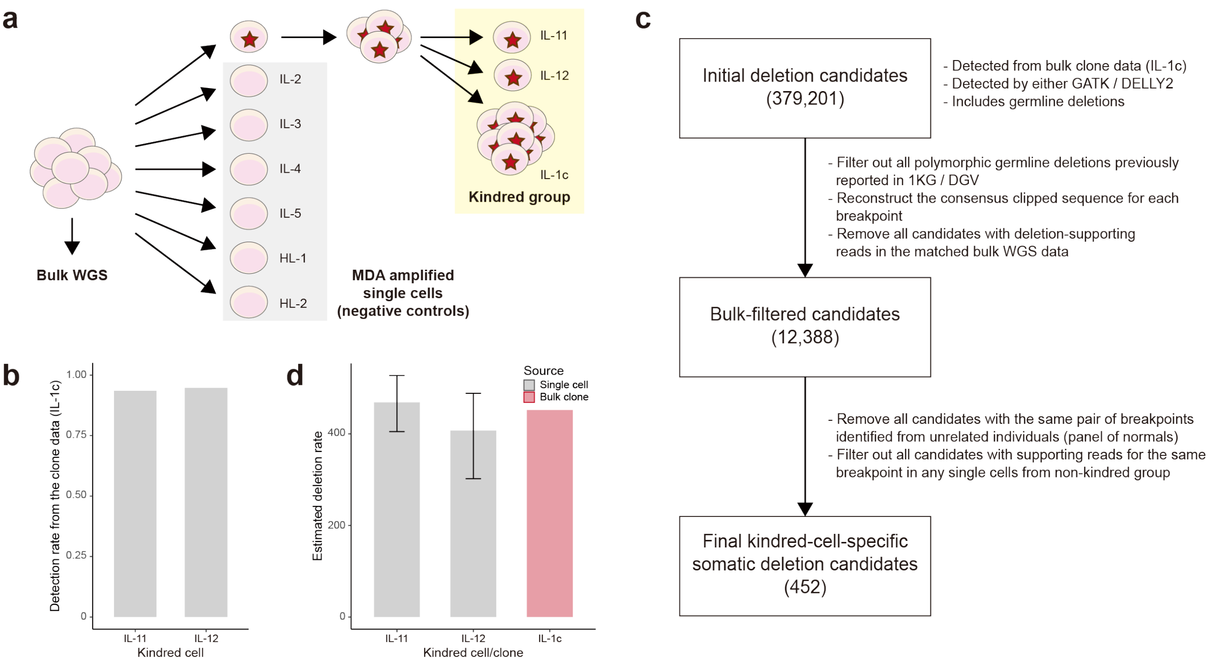

Fig. S3. Performance assessment of PhaseDel using public kindred clone data. (a) Schematic of experimental design for kindred cell system from Dong et al[2]. Two single cells (IL-11, IL-12) and one kindred clone (IL-1c) share the same somatic mutations (red stars) derived from a single parent cell. Single-cell-derived PhaseDel calls that are not observed in the bulk clone are considered as false positives in the assessment. (b) The fraction of single-cell-derived PhaseDel deletions confirmed by unamplified bulk clone WGS data. (c) Filtering process for selecting kindred-cell-specific somatic deletion candidates from the bulk clone data. The final count was considered as the rate estimation by PhaseDel from kindred scWGS data. (d) The comparison between PhaseDel-estimated deletion rates from kindred single cells (grey) and the actual deletion count from the bulk clone (pink). Bar graph, mean±95% CI derived from 10,000 MCMC iterations. Source data are provided as a Source Data file.

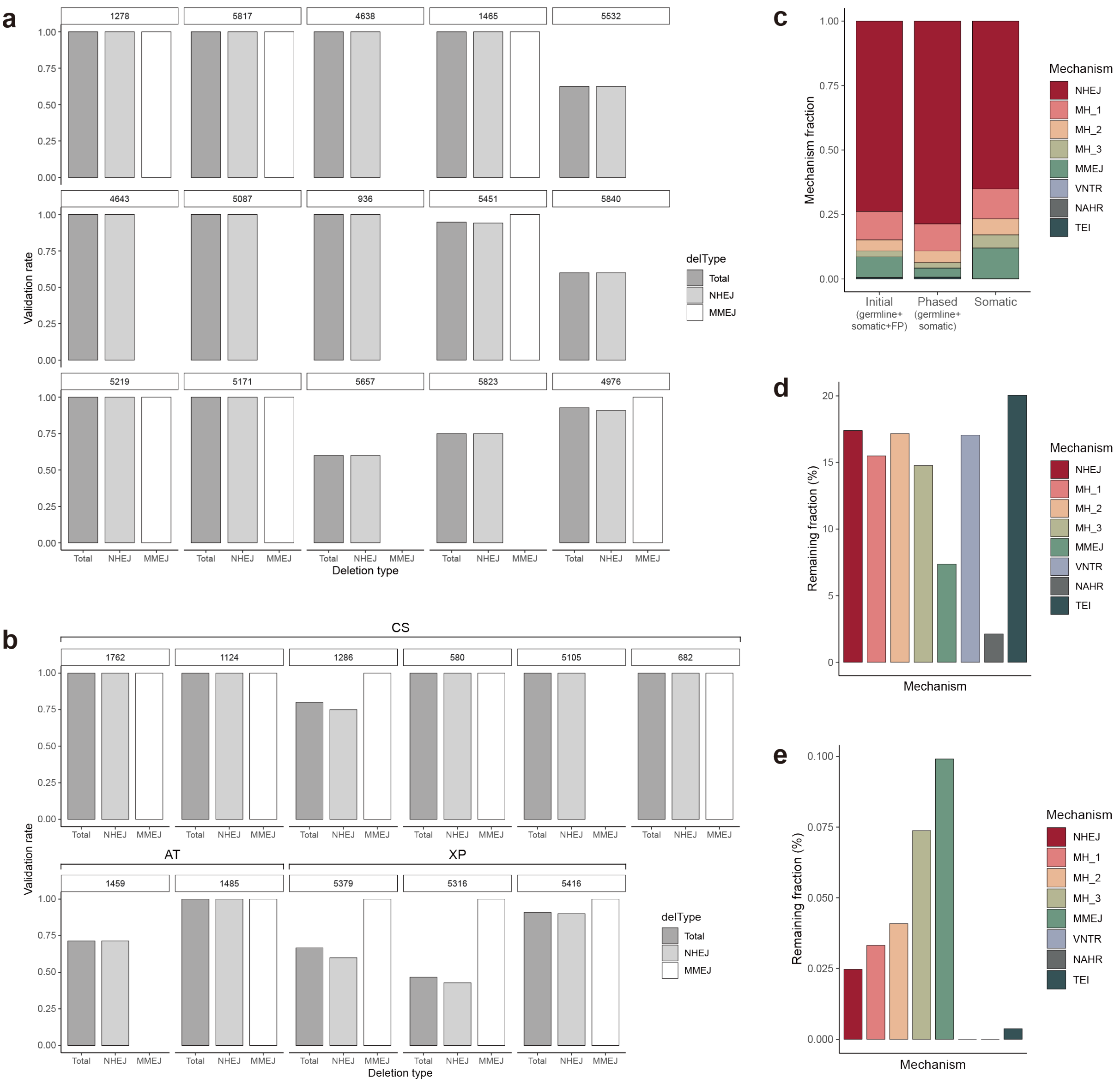

Fig. S4. Validation of selected deletion candidates by ultra-deep amplicon sequencing. (a, b) Validation rates for selected somatic candidates per normal (a) and disease (b) individual. Validation rates for three different groups (total, NHEJ, MMEJ) were measured and depicted. Two normal individuals (5559, 5943) who failed to generate target amplicons due to quality issues are excluded from the figure. An absence of a bar (e.g. MMEJ in 5532) represents the absence of corresponding type of candidates in the validation. (c) The relative fraction of each mechanism out of all deletion candidates at three major steps (collecting initial deletion candidates, removing artifactual candidates through linkage analysis, selecting somatic candidates). (d, e) The remaining fraction of deletion candidates for each mechanism type after the linkage analysis (d) and somatic filtering (e) processes. Source data are provided as a Source Data file.

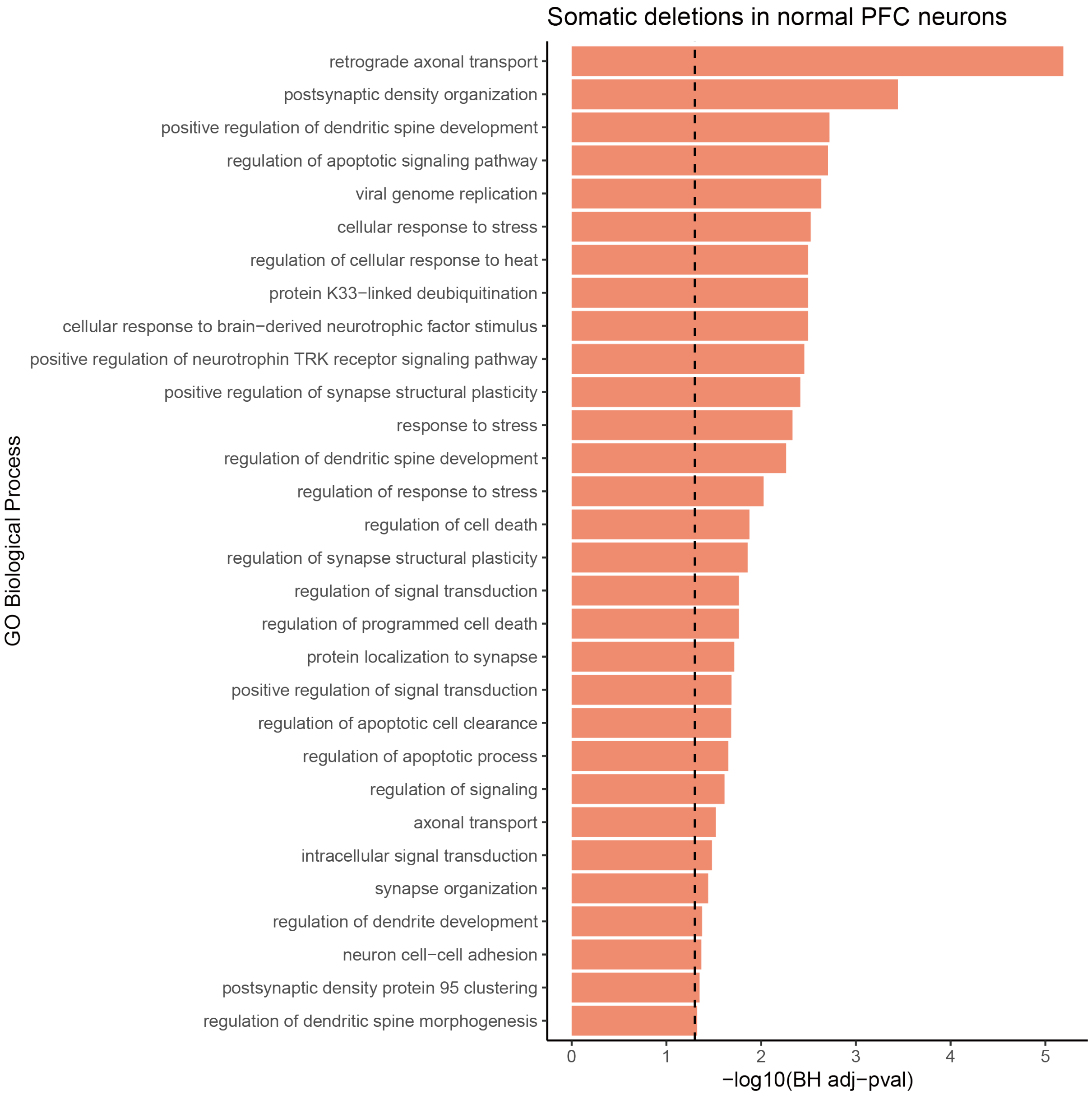

Fig. S5. Neuronal gene ontology (GO) terms enriched for somatic deletions in normal PFC neurons. One-sided binomial test was used to account for gene and deletion size differences using GREAT tool[3] (See Methods). Significantly enriched GO terms (FDR-adjusted p-value<0.05) involved in neuronal functions were selected and shown here. Full list of enriched terms with FDR-adjusted p-value<0.05 is described in Supplementary Data 2. Source data are provided as a Source Data file.

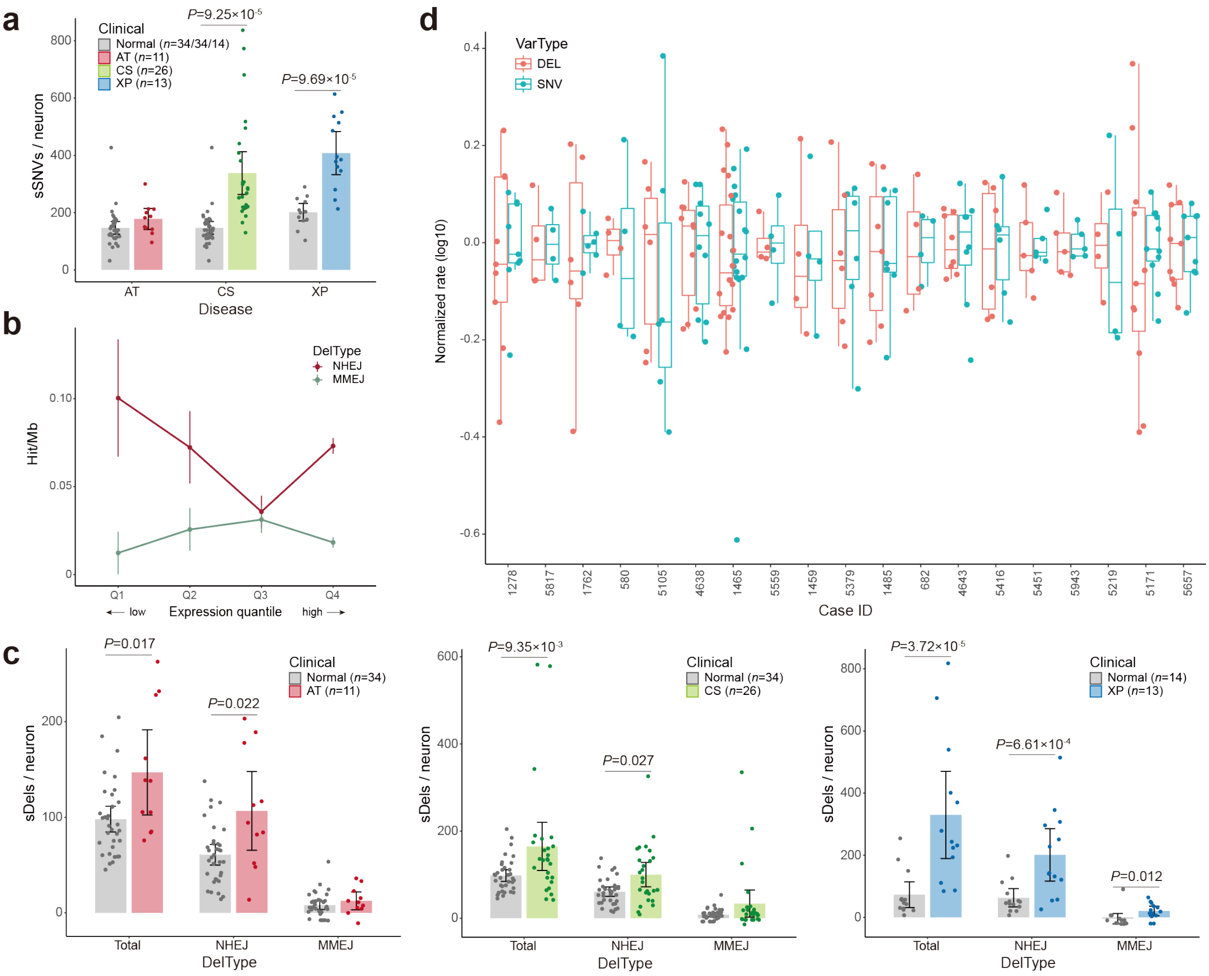

Fig. S6. Comparison of the burdens of somatic SNVs (sSNV) and deletions in single neurons. (a) sSNV burden in AT, CS, and XP single neurons compared to age-matched controls. sSNV rates were estimated using LiRA, a PhaseDel-like phasing method for SNV rate estimation[4]. Note that AT neurons that have DSB repair defects did not present an increase in sSNV burden, whereas the other two diseases with defective nucleotide excision repair presented significant increase in sSNV burden. n, number of single neurons; bar graph, mean±95% CI; two-sided Mann-Whitney U test. (b) Absence of correlation between somatic deletion burden in CS neurons and gene expression levels in normal individuals. Gene expression data from normal PFCs were obtained from the GTEx database. By contrast, figure 4d showed increased NHEJ-deletion burden with the gene expression levels derived from iPSC-derived neural stem cells from CS patients, suggesting that CS mutations cause significant gene expression change and CS neurons also have NHEJ-specific transcription-associated burden in line with the results in normal PFC neurons. *n*=1,000 bootstrap deletion sets; mean±SEM. (c) Age-corrected somatic deletion burden in AT, CS, and XP single neurons compared to age-matched controls. Age effect was corrected by subtracting the predicted age-associated burden using the estimated regression coefficient for normal cells (see Methods). Note that age-corrected MMEJ burdens in normal cells matched in age with XP cells had negative values, because the original MMEJ burdens were lower than the estimated regression line. *n*, number of single neurons; bar graph, mean±95% CI; two-sided Mann-Whitney U test. (d) The dispersion of the estimated rates of sSNVs and deletions in single neurons. Each value was normalized by the corresponding mean of each individual to make a fair comparison. Individuals with ≤ 3 neurons were omitted to adequately show the distribution using boxplots. The number of single neurons per individual is listed in Table S1. Center line, median; box limits, upper and lower quartiles; whiskers, 1.5x interquartile range; points, outliers. Source data are provided as a Source Data file.

Supplementary Tables

Table S1. Case information and number of neurons analyzed in this study

Case ID	Age (years)	Sex	Diagnosis	Reaction buffer used in MDA	Number of neurons
1278	0.4	M	Normal	Epicentre	9
5817	0.7	M	Normal	Epicentre	4
4638	15.1	F	Normal	Epicentre	10
1465	17.5	M	Normal	Epicentre	18
5532	18.4	M	Normal	Epicentre	2
5559	19.8	F	Normal	Epicentre	4
4643	42.2	F	Normal	Epicentre	8
5087	44	M	Normal	Epicentre	3
936	49.2	F	Normal	Epicentre	3
5451	57	F	Normal	Qiagen	5
5943	69	M	Normal	Qiagen	5
5840	75.3	M	Normal	Epicentre	2
5219	77	F	Normal	Epicentre	4
5171	79.2	M	Normal	Epicentre [1-4] / Qiagen [5-11]	11
5511	80.2	F	Normal	Epicentre	3
5657	82.2	M	Normal	Epicentre [1-5] / Qiagen [6-10]	10
5823	82.7	F	Normal	Epicentre	3
4976	104	F	Normal	Qiagen	3
1459	19.9	F	AT	Epicentre	4
1485	24.9	F	AT	Epicentre	7
1762	4.4	F	CS (CSB)	Epicentre	6
1124	4.7	F	CS (CSB)	Epicentre	3
1286	5.8	M	CS (CSB)	Epicentre	3
580	8.4	F	CS (CSB)	Epicentre	4
5105	8.7	M	CS (CSB)	Epicentre	6
682	32.8	M	CS (CSB)	Epicentre	4
5379	24	F	XP (XPA)	Epicentre	6
5316	44.5	F	XP (XPA)	Epicentre	1
5416	46	F	XP (XPD)	Epicentre	6
Total	29 subjects			157 PFC neurons	

Supplementary References

1. Yang L*, et al.* Diverse mechanisms of somatic structural variations in human cancer genomes. *Cell* **153**, 919-929 (2013).

2. Dong X*, et al.* Accurate identification of single-nucleotide variants in whole-genome-amplified single cells. *Nat Methods* **14**, 491-493 (2017).

3. McLean CY*, et al.* GREAT improves functional interpretation of cis-regulatory regions. *Nat Biotechnol* **28**, 495-501 (2010).

4. Bohrson CL*, et al.* Linked-read analysis identifies mutations in single-cell DNA-sequencing data. *Nat Genet* **51**, 749-754 (2019).
