# Supplementary material for: Prevalence and mechanisms of somatic deletions in single human neurons during normal aging and in DNA repair disorders
Source: Nat Commun. 2022 Oct 7;13:5918. doi: 10.1038/s41467-022-33642-w (PMC9546902; doi:10.1038/s41467-022-33642-w)
Supplement: Supplementary file 3 — Description of Additional Supplementary Files [file 41467_2022_33642_MOESM3_ESM.pdf]

File name: Supplementary Data 1

Description: Estimated somatic deletion rate per neuron

File name: Supplementary Data 2

Description: Gene ontology terms enriched for somatic deletions

File name: Source Data Fig. 1

Description: Includes source data for figures 1b, d, e.

File name: Source Data Fig. 3

Description: Includes source data for figures 3a, b, c, d, e.

File name: Source Data Fig. 4

Description: Includes source data for figures 4b, c, d, e.

File name: Source Data Fig. S1

Description: Includes source data for supplementary figures 1a, b, c, d, e, f.

File name: Source Data Fig. S3

Description: Includes source data for supplementary figures 3b, d.

File name: Source Data Fig. S4

Description: Includes source data for supplementary figures 4a, b, c, d, e.

File name: Source Data Fig. S5

Description: Includes source data for supplementary figure 5.

File name: Source Data Fig. S6

Description: Includes source data for supplementary figures 6a, b, c, d.
