# Supplementary material for: Prevalence and mechanisms of somatic deletions in single human neurons during normal aging and in DNA repair disorders
Source: Nat Commun. 2022 Oct 7;13:5918. doi: 10.1038/s41467-022-33642-w (PMC9546902; doi:10.1038/s41467-022-33642-w)
Supplement: Supplementary file 6 — Reporting Summary [file 41467_2022_33642_MOESM6_ESM.pdf]

nature portfolio

Corresponding author(s): Eunjung Alice Lee

Last updated by author(s): Sep 11, 2022

Reporting Summary

Nature Portfolio wishes to improve the reproducibility of the work that we publish. This form provides structure for consistency and transparency in reporting. For further information on Nature Portfolio policies, see our Editorial Policies and the Editorial Policy Checklist.

Statistics

For all statistical analyses, confirm that the following items are present in the figure legend, table legend, main text, or Methods section.

n/a	Confirmed	
☐	☒	The exact sample size (*n*) for each experimental group/condition, given as a discrete number and unit of measurement
☐	☒	A statement on whether measurements were taken from distinct samples or whether the same sample was measured repeatedly
☐	☒	The statistical test(s) used AND whether they are one- or two-sided<br>*Only common tests should be described solely by name; describe more complex techniques in the Methods section.*
☐	☒	A description of all covariates tested
☐	☒	A description of any assumptions or corrections, such as tests of normality and adjustment for multiple comparisons
☐	☒	A full description of the statistical parameters including central tendency (e.g. means) or other basic estimates (e.g. regression coefficient) AND variation (e.g. standard deviation) or associated estimates of uncertainty (e.g. confidence intervals)
☐	☒	For null hypothesis testing, the test statistic (e.g. *F*, *t*, *r*) with confidence intervals, effect sizes, degrees of freedom and *P* value noted<br>*Give P values as exact values whenever suitable.*
☐	☒	For Bayesian analysis, information on the choice of priors and Markov chain Monte Carlo settings
☒	☐	For hierarchical and complex designs, identification of the appropriate level for tests and full reporting of outcomes
☐	☒	Estimates of effect sizes (e.g. Cohen's *d*, Pearson's *r*), indicating how they were calculated

Our web collection on statistics for biologists contains articles on many of the points above.

Software and code

Policy information about availability of computer code

Data collection	Sequencing data was processed to generate analysis-ready BAM using BWA-mem (0.7.17), Picard (2.8.0), and GATK (3.5) as described in Methods section.
Data analysis	GATK (3.5) and DELLY2 (0.7.7) were used to detect initial deletion candidates for linkage analysis. The implemented program for linkage analysis (PhaseDel) and its source code are available at https://sourceforge.net/projects/phasedel/. Gene ontology (GO) enrichment analysis of somatic deletions was performed using rGREAT tool (1.28.0). All statistical tests including linear regression were performed using R software (version 4.0.1). Gene expression levels of GTEx data were measured while controlling for age and gender using DESeq2 (1.24.0). ANNOVAR (2017Jul17) was used to annotate the genomic region and the affected genes for somatic deletion candidates.

For manuscripts utilizing custom algorithms or software that are central to the research but not yet described in published literature, software must be made available to editors and reviewers. We strongly encourage code deposition in a community repository (e.g. GitHub). See the Nature Portfolio guidelines for submitting code & software for further information.

Data

Policy information about availability of data

All manuscripts must include a data availability statement. This statement should provide the following information, where applicable:

- Accession codes, unique identifiers, or web links for publicly available datasets
- A description of any restrictions on data availability
- For clinical datasets or third party data, please ensure that the statement adheres to our policy

Single-neuron whole-genome sequencing data of control individuals and of individuals with CS and XP have been deposited in the NCBI Sequence Read Archive

(SRP041470, SRP061939), the NIH Alzheimer's disease genomic data repository (NIAGADS; NG00121) and dbGAP (phs001485.v1.p1). Single-neuron whole-genome sequencing data of AT patients and targeted amplicon sequencing data for validation of selected deletion candidates are deposited in dbGaP (phs003005.v1.p1).

Field-specific reporting

Please select the one below that is the best fit for your research. If you are not sure, read the appropriate sections before making your selection.

☒ Life sciences ☐ Behavioural & social sciences ☐ Ecological, evolutionary & environmental sciences

For a reference copy of the document with all sections, see nature.com/documents/nr-reporting-summary-flat.pdf

Life sciences study design

All studies must disclose on these points even when the disclosure is negative.

Sample size	In order to maximize power, we utilized all available single-cell whole-genome sequencing (scWGS) data including previously published data sets (Lodato et al, Science, 2017). scWGS data for 107, 26, 13, and 11 PFC single neurons from 18 neurotypical individuals, six CS, three XP, and two AT patients were included. Sample sizes (number of sequenced cells) for newly generated data were chosen commensurate with the numbers necessary to detect effects in a previous study of normal aging (Lodato et al, Science, 2017). All group comparisons between disease and age-matched normal neurons showed significant differences.
Data exclusions	15 single cells that failed to estimate the deletion rate using PhaseDel due to excessive whole-genome amplification errors were excluded (13, 1, and 1 from normal, CS, and XP cells, respectively) as described in the manuscript.
Replication	To improve reproducibility, we performed experiments on multiple neurons from each case. To validate somatic deletion candidates identified by PhaseDel, a total of 244 somatic candidates were randomly selected across all individuals and tested using independent ultra-deep amplicon sequencing method. 209/244 (85.7%) candidates were validated with the predicted breakpoints.
Randomization	Not relevant to our study since we utilized all available data sets without any allocation of samples.
Blinding	Blinding was not relevant. All the data were processed using the same computational procedure.

Reporting for specific materials, systems and methods

We require information from authors about some types of materials, experimental systems and methods used in many studies. Here, indicate whether each material, system or method listed is relevant to your study. If you are not sure if a list item applies to your research, read the appropriate section before selecting a response.

Materials & experimental systems

n/a	Involved in the study	
☐	☒	Antibodies
☒	☐	Eukaryotic cell lines
☒	☐	Palaeontology and archaeology
☒	☐	Animals and other organisms
☐	☒	Human research participants
☒	☐	Clinical data
☒	☐	Dual use research of concern

Methods

n/a	Involved in the study	
☒	☐	ChIP-seq
☒	☐	Flow cytometry
☒	☐	MRI-based neuroimaging

Antibodies

Antibodies used	anti-NeuN (Millipore, MAB377X, clone A60, AlexaFluor-488 conjugated , 1:1250)
Validation	Reactivity validated by the company for human.

Human research participants

Policy information about studies involving human research participants

Population characteristics	De-identified post-mortem human brain tissue from two females with Ataxia telangiectasia was obtained from the University of Maryland Brain and Tissue Bank of the NIH Neurobiobank. Otherwise, publicly available de-identified human genomic data from previously published literature were analyzed [from 18 control individuals without neurological disease(spanning ages 0.4-104, mean of 44.8, with 9 males and 9 females), six individuals with Cockayne syndrome (spanning ages 4.4-32.8, mean of 10.6, with 3 males and 3 females), and three individuals with Xeroderma pigmentosum (spanning ages 24-45.9, mean of 35.7, with 3 females).

Recruitment	Postmortem brain tissue was obtained from participants in brain donation programs at the UMB NIH Neurobiobank.
Ethics oversight	Research performed on samples of human origin was conducted with Category 4 exemption, as approved by the institutional review board of Boston Children's Hospital (protocol S07-02-0087). The University of Maryland Brain and Tissue Bank conducted the recruitment and consent for these subjects according to their IRB approved protocol, and made the tissues available via the NIH Neurobiobank (NBB). The NBB ethical practices and statement of informed consent can be found here: https://neurobiobank.nih.gov/about-best-practices/

Note that full information on the approval of the study protocol must also be provided in the manuscript.
